# Comparative analysis of the intestinal bacterial community and expression of gut immunity genes in the Chinese Mitten Crab (*Eriocheir sinensis*)

**DOI:** 10.1186/s13568-018-0722-0

**Published:** 2018-12-13

**Authors:** Jing Dong, Xiaodong Li, Ruiyang Zhang, Yingying Zhao, Gaofeng Wu, Jinling Liu, Xiaochen Zhu, Lin Li

**Affiliations:** 1 Liaoning Provincial Key Laboratory of Zoonosis, College of Animal Science & Veterinary Medicine, Shenyang Agricultural University, Shenyang, Liaoning 110866 People’s Republic of China; 2 Research and Development Center, Panjin Guanghe Crab Industry Co., Ltd., Panjin, China

**Keywords:** *Eriocheir sinensis*, Intestinal bacterial community, Gut immunity, Antimicrobial peptides, Gene expression

## Abstract

Remarkably little information is available about the interaction between the gut microbiota and intestinal immunity in fish and crustaceans. In our study, we used Illumina MiSeq sequencing and real-time quantitative PCR to compare the microbial community and immunity genes expression in the foregut, midgut and hindgut of Chinese mitten crab (*Eriocheir sinensis*). Our results indicated that the community richness of the midgut is higher than in the foregut or the hindgut, although the bacterial diversity in the hindgut is higher. The predominant phyla were *Tenericutes* and *Firmicutes* in the foregut, *Tenericutes* and *Proteobacteria* in the midgut and *Proteobacteria*, *Tenericutes* and *Bacteroidetes* in the hindgut. When compared with the midgut, the expression of antimicrobial peptides (AMPs) were significantly elevated in the hindgut (*P *< 0.05), and the gene expression of *EsRelish* (*IMD* pathway) was higher than the *Toll* signaling pathway genes. *Actinobacteria* and *Lactobacillus* had negative correlationship with the expression of AMPs, although *Acinetobacter*, *Bacteroides*, *Flavobacterium* can up-regulate the expression of AMP genes. Collectively, our data indicate that microbiota are site-specific within the digestive tracts of crabs and the bacterial community and intestinal immunity have a close relationship in *E. sinensis*.

**Electronic supplementary material:**

The online version of this article (10.1186/s13568-018-0722-0) contains supplementary material, which is available to authorized users.

## Introduction

The connections between gut flora and host was one of the most important impactors influence aquatic animal health (Chaiyapechara et al. 2012; Yan et al. 2012). The intestinal flora is vital to the development (Shang et al. 2017), immunity and disease resistance of gut (Cerf-Bensussan and Gaboriau-Routhiau 2010). The digestive tract is the richest part of human body in terms of number and diversity of bacterial species (Quigley 2013). The dysregulation of intestinal bacteria was closely related to chronic inflammatory and epithelial barrier dysfunction and other illnesses (Abreu 2010; Olmos et al. 2010). There is a lot of information and fast growing about this topic. Comparative analysis of the intestinal bacterial community and expression of gut immunity genes is very important.

In vertebrates and invertebrates, antimicrobial peptides (AMPs) play a crucial role in gut immunity (Destoumieux-Garzón et al. 2016; Zhang and Gallo 2016) and form effective defense mechanisms against multitudinous pathogens. Many studies on AMPs and invertebrate gut defense have been performed in insects, especially *Drosophila melanogaster*, because this species is the model organism of invertebrate (Broderick 2016; Loch et al. 2017; Rolff and Schmid-Hempel 2016). The key role of AMPs in gut immune response is demonstrated through oral reactive oxygen species (ROS) blockers to *Drosophila* mutants that are defective in the NF-κB pathway, resulting in increased mortality (Ryu et al. 2006). This mortality rate decreases when a single AMP is expressed in the intestines of these flies (Ryu et al. 2008). In addition, closely regulated AMPs in the gut of *Drosophila* seem to be indispensable for maintaining the homeostasis of the intestinal flora (Ryu et al. 2008). The production of AMPs was affected by the *Toll* and *IMD* pathways in *Drosophila* hemocytes, but mainly by the latter in gut (Garcia-Garcia et al. 2013). As the central components of *Toll* and *IMD* signaling pathways, e.g., *EsTolls*, *EsDorsal*, *EsPelle*, *EsRelish* (a novel NF-κB-like transcription factor), these genes have been proven to participate in the innate immunity of Chinese mitten crab *Eriocheir sinensis* (*Crustacea: Decapoda: Brachyura: Varunidae*) and defense against several pathogens (Li et al. 2010; Ying et al. 2015; Yu et al. 2013a, b). The above genes could function as an adapter protein in *Toll* and *IMD* signaling and regulate the production of crab AMP genes in hemocytes (Mu et al. 2011; Ying et al. 2015; Zhang et al. 2010). Then there were AMP genes in hemocytes have various biological functions in *E. sinensis* (Destoumieux-Garzón et al. 2016). Hence, these AMPs can help the screening of therapeutic or preventive agents for healthy breeding of crabs. However, the function of these very important genes in the crab intestine is not clear. The study of AMPs in gut immunity of crustaceans is rare and it is unclear which signaling pathways regulate the production of AMPs.

The *E. sinensis* is widely cultivated in China and other countries because of rich nutritional value and high economic value (Chen and Zhang 2007). The culture of *E. sinensis* has increased dramatically in the past decade following a rise in demand. Current production in China is 820,000 tons with an output value of more than 50 billion in 2015 (China Fishery Statistics Yearbook 2016). In recent years, frequent outbreaks of diseases have resulted the decreased production and economic losses (Ding et al. 2017). Accordingly, considerable effort has been expended on intestinal health research of *E. sinensis*. In the present study, we hypothesized that different regions of the digestive tract in *E. sinensis* have difference in the intestinal epithelium bacterial communities and that may be related to the different expression of digestive tract immune genes. The goal of our research was to explore the bacterial diversity, gut immunity genes expression and the relationship between the two in the various regions of the *E. sinensis* digestive tract. Our results suggest a role for the dominant bacteria in each region of the digestive tract and provide novel insight into the bacterial community and gut immunity of *E. sinensis*. These data will be helpful to understand the relationships between symbiotic bacteria and the host.

## Materials and methods

### Crab culture and crab intestine collection

Healthy female *E. sinensis* (9.5 ± 0.5) g were collected with permission from Panjin Guanghe Crab Industry Co., Ltd. (Panjin, Liaoning, China) in July 2016. In the laboratory, crabs were fed with formula diets (Shenyang Hefeng Aquatic Feed Co., Ltd., Shenyang, Liaoning, China) in a concrete recirculating aquaculture system with the following conditions: dissolved oxygen > 7.0 mg L^−1^, pH 7.5–8.3, ammonia < 0.2 mg L^−1^, and nitrite < 0.01 mg L^−1^. Following acclimation (1 week), feeding was stopped 24 h prior to handling and sampling to minimize the stress on *E. sinensis*. Because the tissue of the crab digestive tract is very small, 24 crabs were used for the determination of intestinal flora, the accumulation of 8 crabs is a sample and repeat three times for each sample. In addition, 6 crabs were used for the determination of intestinal immune gene expression, the accumulation of 2 crabs is a sample and repeat three times for each sample. First, washed the body surface with sterile water. Second, disinfected the outside of the body with 75% ethanol for 2 min. Third, dissected the crab in the aseptic state to remove the digestive tract and were then divided into three segments (foregut, midgut, hindgut). Samples were put into sterile EP tubes and stored at − 80 °C for analysis of gut microbiota and expression of gut immune gene.

### DNA isolation, PCR amplification and Illumina MiSeq sequencing

The digestive tract samples were taken for DNA extraction (0.25–0.3 g wet weight) using the E.Z.N.A.^®^ Soil DNA Kit (OMEGA Bio-tek, Norcross, GA, US). We used PCR to amplify the V3–V4 regions of the bacterial 16S rRNA gene. The primers used in the present study were 338F (5′-barcode-ACTCCTACGGGAGGCAGCAG-3′) and 806R (5′-GGACTACHVGGGTWTCTAAT-3′) (amplicon length: 468 bp), 20 μL of PCR mixture containing 5× FastPfu buffer (4 μL), 2.5 mM dNTPs (2 μL), 5 μM forward primer (0.8 μL), 5 μM revese primer (0.8 μL), FastPfu Polymerase (0.4 μL), and template DNA (10 ng). 16S rRNA sequencing were conducted on Illumina MiSeq platform. Paired-end sequencing 2 × 300 bp was performed to sequence all libraries using an Illumina MiSeq platform according to standard protocols.

### Illumina MiSeq sequencing and data processing

We used the QIIME (version 1.17 http://qiime.org/) package to conduct sequences processing. UPARSE (version 7.1 http://drive5.com/uparse/) and UCHIME were used respectively to cluster operational taxonomic units (OTUs) with a 97% similarity cutoff and chimera identification. The diversity and richness of bacterial community were identified by the ACE, Chao1, Shannon and Simpson, which were conducted utilizing the Mothur program (version v.1.30.1 http://www.mothur.org/wiki/Schloss_SOP#Alpha_diversity). We used RDP Classifier (http://rdp.cme.msu.edu/) for sequences classification against the silva database (Amato et al. 2013). The Venn diagram and heatmap were illustrated using the R package (http://www.R-project.org/). The raw sequencing data were uploaded into the NCBI Sequence Read Archive database (SRA; http://www.ncbi.nlm.nih.gov/Traces/sra/) under accession SRP110849.

### Metagenome prediction using PICRUSt

The metagenomes prediction were made with PICRUSt (Langille et al. 2013). The gene content was predicted for each sample according to KEGG and EggNOG. In the present study, the predictive function component was divided into level 3 of EggNOG (evolutionary genealogy: Non-supervised Orthologous Groups) database pathways. The richness was predicted according to the phylogenetic, and the assessed value is 0.8.

### Intestinal tissue RNA extraction and RT-qPCR

Total RNA extraction of the digestive tract samples was perform using Trizol^®^ reagent (Invitrogen, Carlsbad, CA). The RNA quality and concentration were analyzed spectrophotometrically using a BioDrop μLITE (BioDrop, Cambridge, UK). Reverse transcription of total RNA (1 μg) was conducted in a 20 μL system using PrimeScript™ RT reagent kit with gDNA Eraser (Takara Bio. Inc., Dalian, China).

The primer sequences for the *Toll* signaling pathway genes [*EsToll2* (GenBank accession No. KC011816), *EsPelle* (GenBank accession No. KP795393), *EsDorsal* (GenBank accession No. KC900086)], *IMD* signaling pathway genes [*EsRelish* (GenBank accession No. GQ871279)], antibacterial peptides [*EsALF1* (GenBank accession No. DQ793214), *EsALF2* (GenBank accession No. GU014699), *EsCrus1* (GenBank accession No. GQ200832), *EsCrus2* (GenBank accession No. GQ200833)], and *Es*-*β*-*Actin* (GenBank accession No. HM053699) are described in Additional file 1: Table S1.

All of the primers were synthesized by GenScript Biotechnology Co. Ltd. (Nanjing, China). RT-qPCR was performed employing the LightCycler 480 II system (Roche Applied Science, Branford, CT, USA) in conjunction with SYBR green dye, and the following programme: 95 °C for 30 s, then 45 cycles of 95 °C for 5 s and 60 °C for 40 s, then 1 cycle of 95 °C for 5 s, 60 °C for 60 s, and 95 °C, and terminated at 50 °C for 30 s. The 20 μL reaction mixture consisted of 10 μL of 2× SYBR Premix Ex Taq II (Takara Bio. Inc., Dalian, China), 1.0 μL of each primer and 1.5 μL of cDNA. Gene expression levels were compared with those of *Es*-*β*-*Actin*, and the 2^−∆∆Ct^ method was adopted to data analysis using the light cycler 480 software.

### Statistical analyses

The statistical analyses were performed using the SPSS 17.0 software package (SPSS Inc., Chicago, USA). In the various regions of the *E. sinensis* digestive tract was regarded as the fixed effect. Values were expressed as the mean ± SEM. The diversity of digestive tract bacterial community and the relative mRNA expression of eight gut immune genes (*EsToll2*, *EsPelle*, *EsDorsal*, *EsRelish*, *EsALF1*,*2* and *EsCrus1*,*2*) in the same regions of the *E. sinensis* intestinal tract were compared using a one-way analysis of variance (ANOVA), followed by Bonferroni’s multiple comparisons post hoc test. COG function classification in the intestinal microbiota and the relative mRNA expression of gut immune gene in the intestinal tract of *E. sinensis* were compared using Independent-Samples *T* test. Significance was declared at *P *< 0.05. Principal coordinates analysis (PCoA) based on UniFrac distances were performed to evaluate the overall differences in the gut bacterial community (Jiang et al. 2013). Student’s *t* test (equal variance) was used to analyse bacterial communities in the intestinal tract samples. Spearman coefficient was used for correlation detection.

## Results

### Bacterial composition determined by MiSeq sequencing

Microbial community of the digestive tract was determined using Illumina MiSeq. 32128 sequences were obtained by quality screening for alpha-diversity research. Rarefaction analysis was shown in Additional file 1: Fig. S1. Microbial composition was counted by the proportion of OTUs. Assessment of microbial diversity in the foregut, midgut and hindgut of crabs were completed using α-diversity indexes. The ACE and Chao values were significantly increased in the midgut (*P *< 0.05). Microbial composition in the hindgut were higher α-diversity indexes than those in the midgut or foregut, as revealed through the Shannon and Simpson indexes (Additional file 1: Table S2).

### Microbial community compositions

At the phylum level, a total of 16 phyla were detected in the crab digestive tract, and result showed 12 phyla were present in all three groups (Additional file 1: Fig. S2A). At the genus level, there were 109, 161 and 112 taxa in the foregut, midgut and hindgut groups, respectively (Additional file 1: Fig. S2B).

**Fig. 1:**
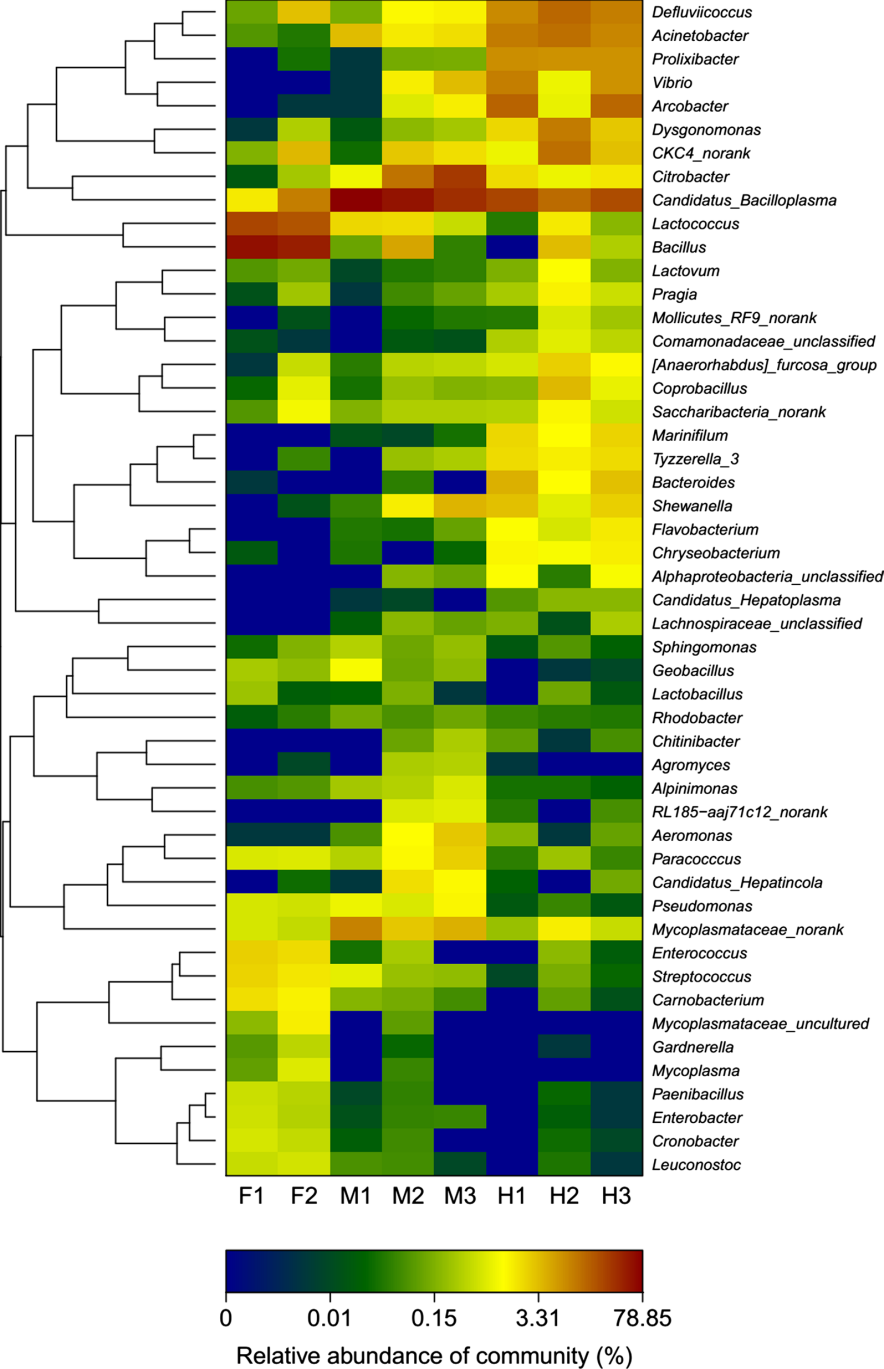
Heatmap of the dominant digestive flora at the genus level. *F* foregut, *M* midgut, *H* hindgut

At the phylum level of the digestive tracts (F, M, H) of all the crabs, *Tenericutes* were the most (33.00%), then *Firmicutes* (29.48%), *Proteobacteria* (28.36%), and *Bacteroidetes* (7.87%). Within the intestines (M, H) of all the crabs, *Tenericutes* were the largest number (41.48%), then *Proteobacteria* (36.18%), *Firmicutes* (10.65%), and *Bacteroidetes* (10.35%) (Additional file 1: Fig. S3). At the genus level of the digestive tract (F, M, H), the dominant genera were composed of *Bacillus* (14.53%), *Lactococcus* (6.28%), *Citrobacter* (6.02%), *Acinetobacter* (4.88%), *Rhodospirillaceae* (4.78%), *Arcobacter* (4.06%), *Erysipelotrichaceae* (3.18%), *Vibrio* (2.89%), *Prolixibacter* (2.74%), *Mycoplasmataceae* (2.47%), *Dysgonomonas* (2.06%), *Shewanella* (1.55%), and *Bacteroides* (1.13%). Within the intestines (M, H), the dominant genera were composed of *Candidatus Bacilloplasma* (38.07%), *Citrobacter* (7.97%), *Acinetobacter* (6.48%), *Rhodospirillaceae* (5.80%), *Arcobacter* (5.41%), *Vibrio* (3.85%), *Prolixibacter* (3.65%), *Erysipelotrichaceae* (3.60%), *Mycoplasmataceae* (2.84%), *Dysgonomonas* (2.68%), *Shewanella* (2.06%), *Bacillus* (1.55%), *Bacteroides* (1.50%), *Lactococcus* (1.17%), *Tyzzerella_3* (1.09%), and *Marinifilum* (1.01%) (Additional file 1: Fig. S4). The top fifty bacterial taxa are represented in a heat map (Fig. 1).

### Microbial community similarity among foregut, midgut and hindgut

**Fig. 2:**
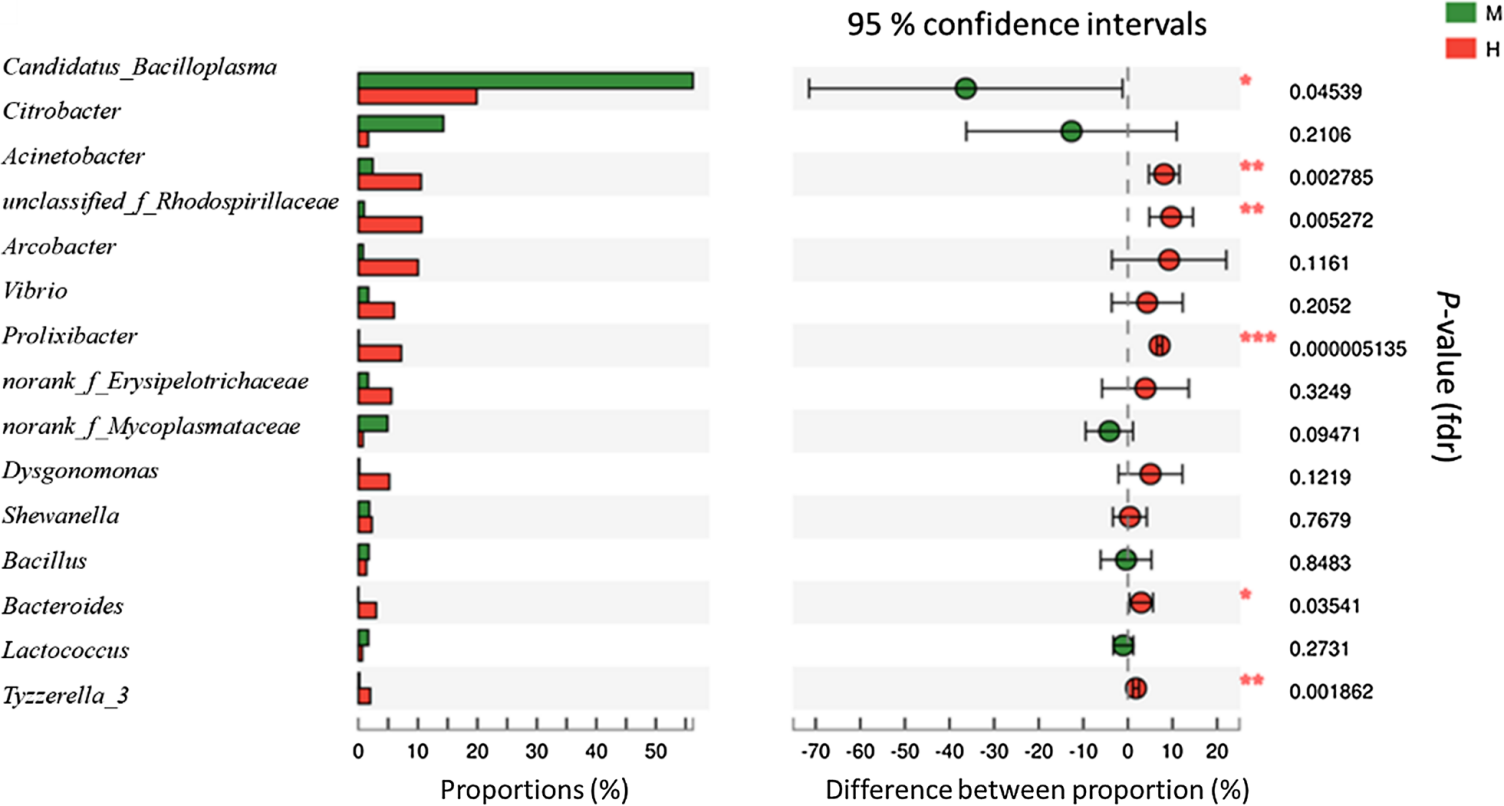
Student’s *t*-test bar plot on genus level of bacterial communities in the intestinal tract samples. The ordinate indicates the name of the genus level, the abscissa represents the percentage of the abundance of the sample. The color of the bar represents different groups M: midgut (green), H: hindgut (red). 0.01 < *P* ≤ 0.05*, 0.001 < *P* ≤ 0.01**, *P *≤ 0.001***

PCoA showed a clustering of the foregut, midgut and hindgut by PC1 (51.95%) and PC2 (29.6%) of the explained variance (Additional file 1: Fig. S5). At the genus level, the abundance of *Candidatus Bacilloplasma* was 56.24% (midgut) and 19.9% (hindgut), of which the midgut had significantly higher levels than the hindgut (*P* = 0.045) (Fig. 2). By contrast, there was a significantly decreased level of bacteria from the *Acinetobacter* (*P* = 0.003), *unclassified_f_Rhodospirillaceae* (*P* = 0.005), *Prolixibacter* (*P* = 0.000), *Bacteroides* (*P* = 0.035), and *Tyzzerella_3* (*P* = 0.002) taxa in the midgut (Fig. 2).

### Metagenome prediction of gut microbiota

**Fig. 3:**
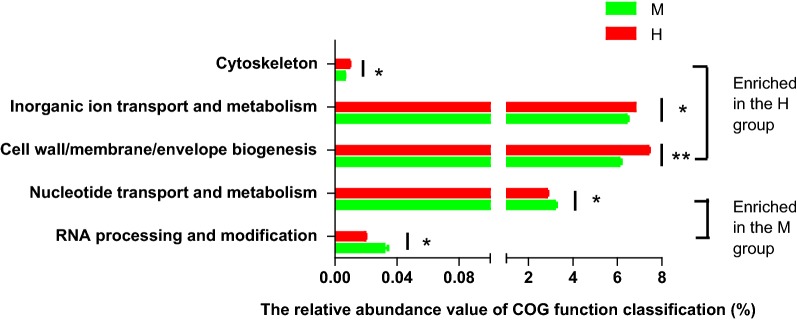
COG function classification in the microbiota of the intestinal tract samples. M: midgut (green), H: hindgut (red). 0.01 < *P* ≤ 0.05*, 0.001 < *P* ≤ 0.01**

A total of 25 gene families were identified in all samples, five function classification were significantly differentially rich between the midgut and the hindgut (*P *< 0.05) (Fig. 3). Compared with the hindgut, the midgut had a higher relative abundance of nucleotide transport and metabolism (*P *= 0.034) and RNA processing and modification (*P *= 0.032) gene families, and a lower relative abundance of cytoskeleton (*P *= 0.025), inorganic ion transport and metabolism (*P *= 0.008), and cell wall/membrane/envelope biogenesis (*P *= 0.000) gene families.

### Gene expression in the intestinal tract

**Fig. 4:**
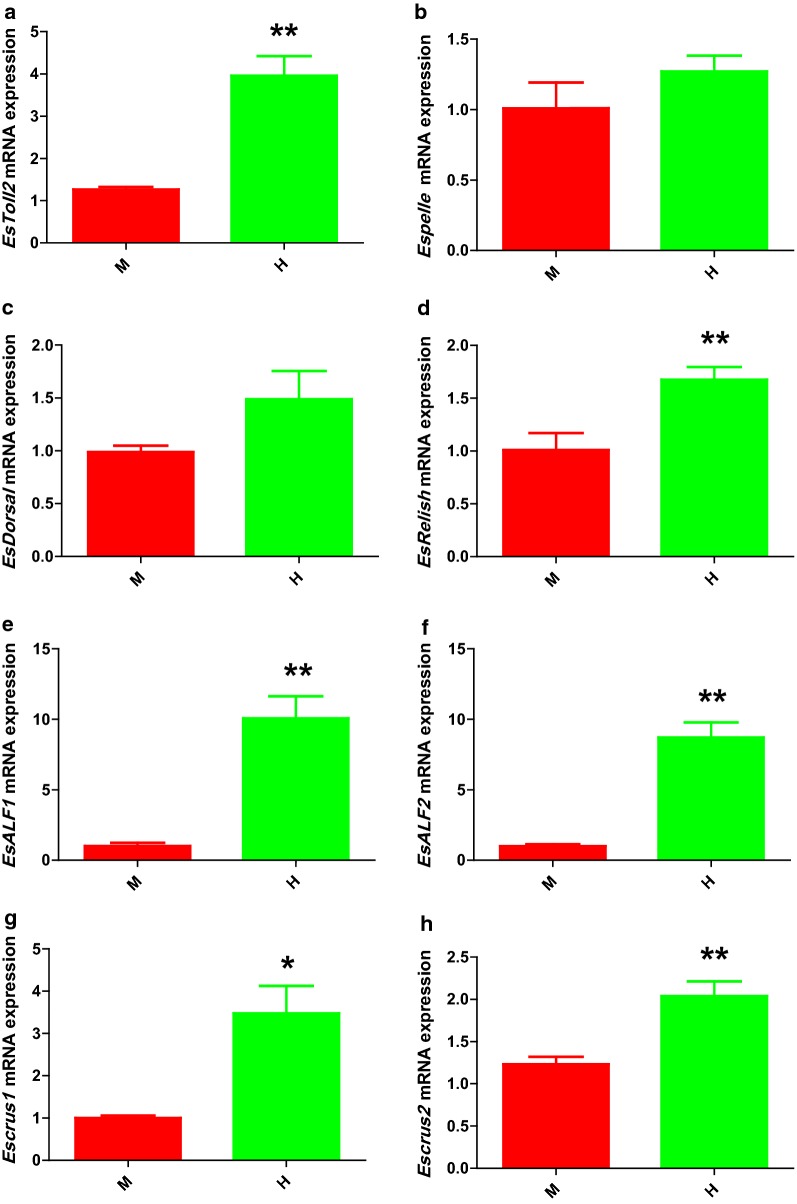
Comparative analysis on gut immune gene expression (relative to *Es*-*β*-*Actin*) in the intestinal tract of *E. sinensis* (mean ± SEM, n = 3).** a**–**h**: relative expression profile of 8 immune-related genes (*EsToll2*,* EsPelle*,* EsDorsal*,* EsRelish*,* EsALF1*,* EsALF2*,* EsCrus1*,* EsCrus2*) in crab midgut and the hindgut. M: midgut (red), H: hindgut (green). 0.01 < *P* ≤ 0.05*, *P *≤ 0.01**

**Fig. 5:**
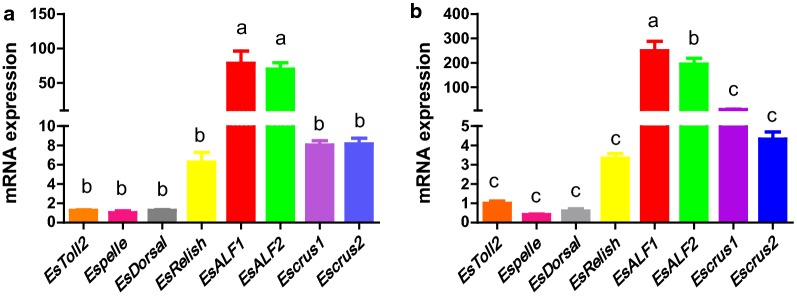
Comparative analysis on eight gut immune genes expression (relative to *Es*-*β*-*Actin*) in the same regions of the *E. sinensis* intestinal tract (mean ± SEM, n = 3). **a** Midgut, **b** hindgut. Different letters indicate significant differences (*P* < 0.05)

The relative mRNA expression of the *Toll* signaling pathway (*EsToll2*, *EsDorsal*, *EsPelle*), *IMD* signaling pathway (*EsRelish*) and AMPs (*EsALF1*, *EsALF2*, *EsCrus1*, *EsCrus2*) were detected by real-time quantitative polymerase chain reaction (RT-qPCR). Compared with the midgut, *EsToll2* (*P *= 0.008), *EsRelish* (*P *= 0.005), *EsALF1* (*P *= 0.001), *EsALF2* (*P *= 0.006), *EsCrus1* (*P *= 0.021), and *EsCrus2* (*P *= 0.002) gene expression were significantly up-regulated in the hindgut (*P *< 0.05). However, *EsDorsal* (*P *= 0.077) and *EsPelle* (*P *= 0.102) mRNA expression levels were only slightly higher in the hindgut (Fig. 4). In addition, the RT-qPCR analysis showed that the expression changes of eight genes had similar trends in the midgut and the hindgut. The levels of *EsALF1* and *EsALF2* mRNA expression were significantly higher than six other genes in the intestinal tract (*P *< 0.05). *EsRelish*, *EsCrus1* and *EsCrus2* expression levels were higher than those of the *Toll* signaling pathway genes (*EsToll2*, *EsDorsal*, *EsPelle*), but no significant difference was detected (Fig. 5).

### Correlation analysis

**Fig. 6:**
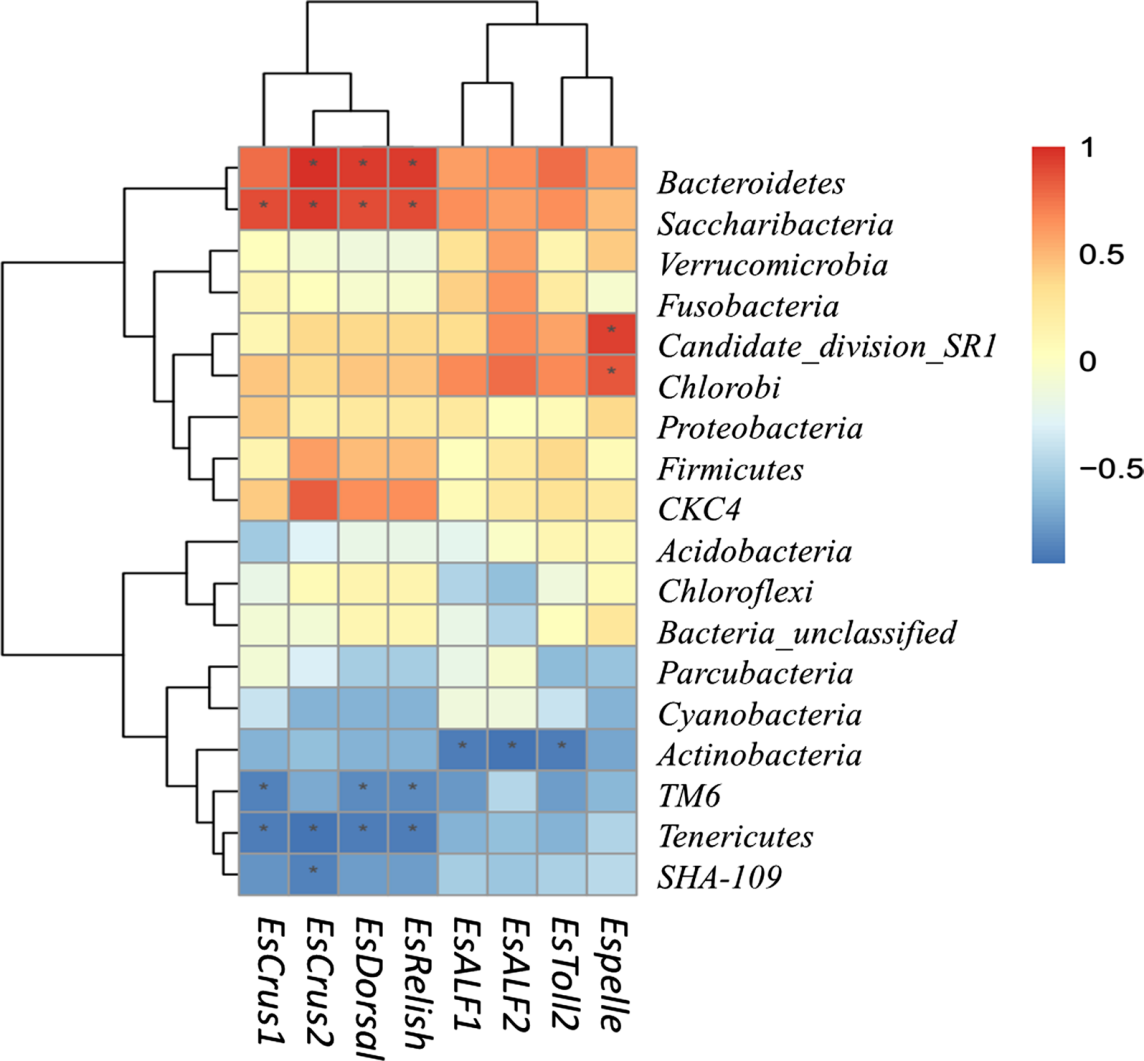
Correlation analyses between intestinal microorganism (phylum level) and gut immune genes expression. R values are correlation coefficient shown in different colors in the figure, the red color represents a positive correlation, the blue color represents a negative correlation. *P < 0.05

The relationships among immune gene expression and intestinal microbiota were evaluated. At the phylum level (Fig. 6), the results showed that *Actinobacteria* negatively correlated with *EsToll2*, *EsALF1*, and *EsALF2* gene expression (*P *< 0.05). *Candidate_division_SR1* and *Chlorobi* and *EsPelle* gene expression were positive correlations (*P *< 0.05). In addition, the abundance of four taxa (tow negative: *TM6*, *Tenericutes*; tow positive: *Bacteroidetes*, *Saccharibacteria*) was related to increase *EsRelish*, *EsCrus1*, *EsCrus2* gene expression (*P *< 0.05). At the genus level (Additional file 1: Fig. S6), *Acinetobacter* positively correlated and *Paracoccus* negatively correlated with the mRNA expression of *EsALF1* (*P *< 0.05). The abundance of nine taxa (six negative: *Defluviimonas*, *Paracoccus*, *Alpinimonas*, *Gemmobacter*, *MNG7_norank*, *Pseudomonas*; three positive: *Chryseobacterium*, *Bacteroides*, *Candidatus Hepatoplasma*) was correlated with increased *EsALF2* gene expression (*P *< 0.05). The abundance of nine taxa (one negative: *Candidatus Bacilloplasma*; eight positive: *Acinetobacter*, *Mollicutes*_*RF9_norank*, *Lactovum*, *Pragia*, *Saccharibacteria*_*norank*, [*Anaerorhabdus*]*_furcosa*_group, *Defluviicoccus*, *Dysgonomonas*) was correlated with increased *EsCrus1* gene expression (*P *< 0.05). The abundance of seventeen taxa (five negative: *Candidatus Bacilloplasma*, *Rhodobacter*, *Gemmobacter*, *MNG7_norank*, *Clostridium_sensu_stricto_1*; twelve positive: *Candidatus Hepatoplasma*, *Mollicutes_RF9_norank*, *Lactovum*, *Pragia*, *Saccharibacteria_norank*, [*Anaerorhabdus*]*_furcosa*_group, *Defluviicoccus*, *Dysgonomonas*, *Comamonadaceae_unclassified*, *Morganella*, *Coprobacillus*, *Sphingobacterium*) was related to increase *EsCrus2* gene expression (*P *< 0.05). The abundance of eight taxa was related to increase mRNA expression of *EsToll2* (*P *< 0.05). The abundance of fourteen taxa was correlated with increased mRNA expression of *EsRelish* (*P *< 0.05), as shown in Additional file 1: Fig. S6.

## Discussion

In the human and mouse gut, *Firmicutes* and *Bacteroidetes* are the dominant phyla (Consortium 2012). In chickens, *Firmicutes* is the most prevalent, followed by *Proteobacteria*, *Bacteroidetes* and *Actinobacteri*a (Choi et al. 2014). In ruminants, *Firmicutes* is the predominant phylum, followed by *Bacteroidetes* and *Proteobacteria* (Ye et al. 2016). In fish, *Proteobacteria* and *Fusobacteria* are the most dominant gut phyla (Clements et al. 2014; Tyagi and Singh 2017). In invertebrates, *Proteobacteria* and *Firmicutes* dominate the gut of *Drosophila melanogaster* (Broderick and Lemaitre 2012). *Tenericutes* and *Proteobacteria* are dominant in *L. vannamei* and *E. sinensis* (Zhang et al. 2014a, 2016). *Proteobacteria* and *Bacteroidetes* are the two dominant populations in *E. sinensis* (Li et al. 2007). Our research results showed that *Firmicutes* and *Tenericutes* were the predominant phyla in the foregut, *Tenericutes* and *Proteobacteria* were predominant in the midgut, and *Proteobacteria*, *Tenericutes* and *Bacteroidetes* were predominant in the hindgut of *E. sinensis*. In crabs, the epithelial surface structure of the midgut and the hindgut is different and therefore so is the bacterial adhesion (Chen et al. 2015). Although the dominant species are similar, the abundances are different, indicating that bacteria in the phyla *Tenericutes*, *Bacteroidetes*, *Firmicutes* and *Proteobacteria* maintain a close relationship with the host (Chen et al. 2015). Our findings were consistent with the above and suggest that there are evolutionary relationships between these animals. The intestinal flora and symbiotic microbes are very similar in crustaceans, although the functions of these symbiotic bacteria remain unknown.

We observed that the ACE and Chao values were significantly increased in the midgut. The microbial composition in the hindgut showed higher α-diversity indexes (Shannon and Simpson) comparing the midgut and the foregut. The gut flora of pond crabs had more inter-subject variation compared to wild crabs (Li et al. 2007). The unweighted UniFrac PCoA and Venn diagram revealed differences between the bacterial communities within the foregut, midgut and hindgut, indicating that the bacterial community and digestive tract function are closely related. These differences may be due to several reasons. The gastrointestinal organizational structure is different in the foregut, midgut and hindgut. Additionally, the various digestive tract regions have different functions; the foregut (stomach) is used to grind food, the midgut is responsible for digestion and absorption, and the hindgut stores stool and defends against microbes.

At the genus level, the abundance of *Candidatus Bacilloplasma* was significantly increased in the midgut compared to the microbes of the hindgut. By contrast, there was a significant decrease in *Acinetobacter*, *unclassified_f_Rhodospirillaceae*, *Prolixibacter*, *Bacteroides*, and *Tyzzerella_3* in the midgut. *Candidatus Bacilloplasma* was a new lineage of bacteria specifically related to the intestinal surface of *Porcellio scaber* (Kostanjsek et al. 2007). They are rod-shaped bacteria that colonize the gut surface of crustacea and are affiliated with the class *Mollicutes*. *Mollicutes* is a common bacterium which is symbiotic and beneficial to the host in the intestinal tract of arthropods (Leclercq et al. 2014). Many of the *Mollicutes*, including *Candidatus Bacilloplasma*, colonize the digestive tract with food (Kostanjsek et al. 2007). Early research showed that there were rich in flora in the hindgut of *Decapoda*, but no absorption of nutrients, therefore, there is no competition with the hosts. Later, there has been an increasing interest on the midgut microbiota of *Decapoda* as suitable part for the research of microorganism–host interactions, and where nutrient absorption takes place (Meziti et al. 2010). However there is limited information comparing results between midgut and hindgut. In the present study, *Candidatus Bacilloplasma* were significantly more abundant in the midgut, which is where food digestion primarily occurs, indicating that the bacterial community and digestive tract function have a close relationship. *Rhodospirillaceae*, which are photosynthetic bacteria and contain abundant nutrients and functional factors (Qiu et al. 2008), were found to be significantly more abundant in the hindgut, suggesting that these bacteria could be potentially used as a probiotic.

The microbiota of the digestive tract are influenced by genetic background, environment and diet, etc. Early research had reported that gut microorganism are very key role in nutrients absorption and degradation (Zhang et al. 2014b), living environments (Huang et al. 2014), and immunity (Ye et al. 2016). Recently, research has aimed to determine the relationship between intestinal microbiota and the host in invertebrates (Xiong et al. 2015; You et al. 2014; Zhang et al. 2014a). Although intestinal microbes had closely related to the immune system, their interaction with gut immunity in invertebrates, especially in crustaceans, remains unknown.

Intestinal flora plays a key role in maintaining host health. In this study, the difference in predictive functional pathways between the midgut and hindgut indicated that change of microbial composition may have the ability to modify their functional capabilities. Our data showed that the genes responsible for nucleotide transport and metabolism and RNA processing and modification were up-regulated in the midgut compared with the hindgut. However, the genes were down-regulated associated with the cytoskeleton, inorganic ion transport and metabolism, and cell wall/membrane/envelope biogenesis. Because there are only a few studies concerning the gut bacterial community of crabs, the biological significance of these distinct differences remains unclear. Inorganic ion transport and metabolism includes phosphate, sulfate, and various cation transporters (Gill et al. 2006). The bacterial proteins identified were classified into COG functional categories, there is more highly represented in inorganic ion transport and metabolism of healthy children compared with obese children with non-alcoholic fatty liver disease (Michail et al. 2015). The increased abundance of cell wall/membrane/envelope biogenesis genes may suggest involvement with transmembrane transport and the exocytosis of antibiotics to resist the effects of tetracycline hydrochloride, indicating that gut microbiota can improve antibiotic resistance capability. Therefore, opportunistic bacteria can survive within the mouse gut (Yin et al. 2015).

There is a wealth of information about the intestinal immune regulation mechanism of vertebrate and its interaction with the symbiotic microbiota on the surface of intestinal mucosa (Rooks and Garrett 2016). However, there is little information concerning the gut immune mechanisms of invertebrates at the barrier epithelia (Garcia-Garcia et al. 2013). The relationship between commensal microbiota and gut immunity of crustaceans is important, although few data are available. We measured the relative mRNA expression of three genes in the *Toll* signaling pathway, one gene in the *IMD* signaling pathway and four AMP genes in the guts of crabs by RT-qPCR. We showed that the mRNA expression levels of *EsToll2*, *EsRelish*, *EsALF1*, *EsALF2*, *EsCrus1*, and *EsCrus2* were significantly up-regulated in the hindgut compared with the midgut. However, the *EsDorsal* and *EsPelle* mRNA expression levels were only slightly higher in the hindgut. These results are consistent with previous reports (Watthanasurorot et al. 2012) and speculated that the hindgut flora has more regulation of intestinal immunity than the midgut flora, and it can better explain the fact that pathogens can enter easily the body through the midgut. In addition, the expression changes of eight genes showed similar trends in the midgut and the hindgut. *EsALF1* and *EsALF2* mRNA expression were significantly higher than the six other genes within the intestinal tract. Expression of *EsRelish*, *EsCrus1* and *EsCrus2* were found to be higher than the *Toll* signaling pathway genes (*EsToll2*, *EsDorsal*, *EsPelle*), although there was no significant difference. As previously reported, the intestinal immunity of amphioxus indicated that the expression of the *TLRs* gene is very low and the response to pathogens is small, which is contrary to vertebrates (Garcia-Garcia et al. 2013). This study revealed different expression of immune genes in the midgut and the hindgut of *E. sinensis*, and different sensitivity to external stimuli, which may be related to its different organizational structure. AMPs (*EsALF1*, *EsALF2*) act a pivotal part in intestinal immunity and may be regulated by the *IMD* pathway. In a study on the black tiger shrimp (*Penaeus monodon*), the digestive organ leads to quite severe injury after oral infection by *Vibrio harveyi*. RT-qPCR analysis showed that *V. harvey* induced up-regulation of AMP (*ALF3*, *crustin* and *penaeidin*) genes expression with infection time in the intestine of juvenile shrimp (Soonthornchai et al. 2010).

Innate immunity is the only host defense mechanism in invertebrates which lacks the high selection mechanisms of adaptive immunity. Recent reports have suggested that *EsToll1* and *EsToll2* are expressed in the digestive tract of *E. sinensis* (Yu et al. 2013a), although their gene expression level is not high. This result is consistent with our findings. Interestingly, *TLRs* were expressed at low levels in the gut of *Drosophila melanogaster*. Many reports have shown that the correlation between the composition of gut-associated bacteria and inflammatory parameters can serve as a biological indicator to evaluate the occurrence of diseases (Newsholme and de Bittencourt Jr 2016; Xiong et al. 2017; Zeng et al. 2017). However, the function and relationship of intestinal microbes and the occurrence of diseases in fish and crustacean are still poorly understood. Our results showed that the abundance of *Actinobacteria* negatively related to the relative mRNA expression of *EsToll2*, *EsALF1*, and *EsALF2*. Previous results showed that marine *Actinobacteria* can produce enzymes and can provide an important niche for probiotics in aquaculture with an increasing demand for both probiotics and prebiotics (Das et al. 2008). Subsequent studies showed that *Actinobacteria* isolated from the intestinal microorganism of two freshwater fish conferred resistance to human and fish pathogens and demonstrated its potential to produce biologically active compounds (Jami et al. 2015). Thus, an *actinobacterial* microarray can reflect the changes in body condition and culture environment (Wang et al. 2017). We speculate that *Actinobacteria* are symbiotic bacteria in the crab intestines and do not increase AMP gene expression. At the genus level, *Acinetobacter*, *Bacteroides*, *Flavobacterium* and 19 other species of bacteria can up-regulate the expression of AMPs to protect against pathogen invasion. Some bacteria, such as *Lactobacillus*, were highly abundant in the intestinal flora but did not have a positive correlation with AMP expression, suggesting that the intestinal mucosal immune system identifies these bacteria as symbiotic bacteria. The use of complex strategies to regulate the NF-κB signaling pathway and to inhibit the expression of AMPs is not clear and requires further research.

In conclusion, this study showed that the foregut, midgut and hindgut of *E. sinensis* have distinct microbiota. Community richness in the midgut was higher than that in the foregut or the hindgut, although the bacterial diversity in the hindgut was higher these data suggest that symbiotic microbiota are site-specific within the digestive tract of crabs. Data from this study also revealed the differences in the bacterial communities of the midgut and the hindgut. RT-PCR revealed that the mRNA expression level of AMPs was significantly up-regulated in the hindgut compared with the midgut and that the gene expression of *EsRelish* (*IMD* pathway) was higher than the *Toll* signaling pathway genes. AMP genes (*EsALF1*, *EsALF2*) play an important role in intestinal immunity and are presumably regulated by the *IMD* pathway. Correlation analysis revealed that the relationship between bacteria in the intestine of *E. sinensis* and the production of AMPs and provide information on the potential probiotics and biological indicators to evaluate the occurrence of disease. Overall, these results provide a comprehensive picture of the bacterial structure of the *E. sinensis* digestive tract and help to further elucidate disease prevention and treatment in crustaceans.

## Additional file

**Additional file 1.** Additional figures and tables.
