# Supplementary material for: Comparative analysis of the intestinal bacterial community and expression of gut immunity genes in the Chinese Mitten Crab (Eriocheir sinensis)
Source: AMB Express. 2018 Dec 13;8:192. doi: 10.1186/s13568-018-0722-0 (PMC6292837; doi:10.1186/s13568-018-0722-0)
Supplement: Supplementary file 1 — Additional file 1. Additional figures and tables. [file 13568_2018_722_MOESM1_ESM.docx]

**Comparative analysis of the intestinal bacterial community and expression of gut immunity genes in the Chinese Mitten Crab (*Eriocheir sinensis*)**

**Jing Dong, Ruiyang Zhang, Yingying Zhao, Gaofeng Wu, Jinling Liu, Xiaochen Zhu, Lin Li*, Xiaodong Li***

Liaoning Provincial Key Laboratory of Zoonosis, College of Animal Science & Veterinary Medicine, Shenyang Agricultural University, Shenyang, Liaoning, 110866, P.R. China.

*Corresponding authors:

Lin Li: lilin619619@163.com, Fax: 86-88487156, Tel.:86-13840169129;

Xiaodong Li: lxd001@ceraap.com, Fax: 86-88487156, Tel.:86-13700072918


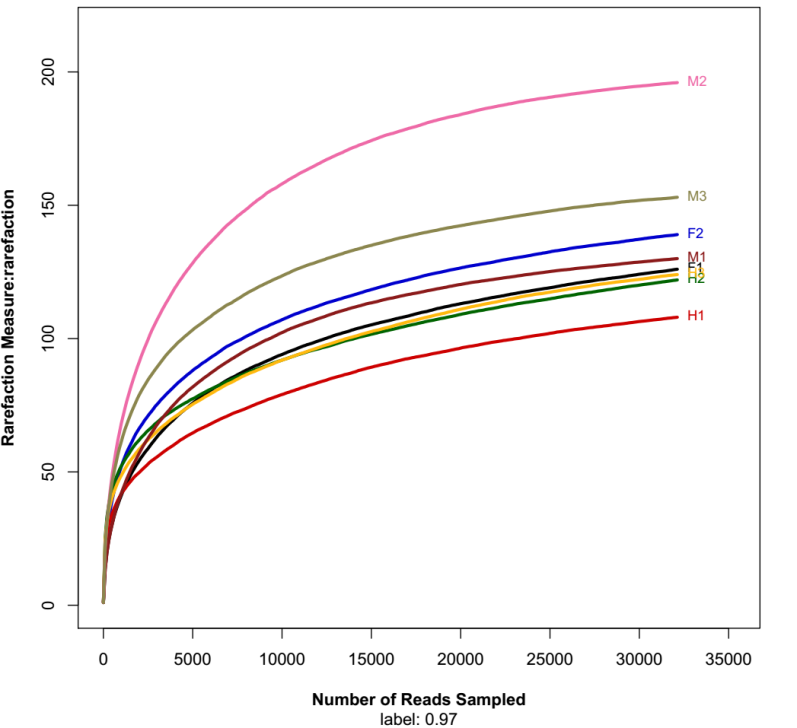


**Fig. S1** Rarefaction analysis of microbiota from foregut (F1, F2), midgut (M1, M2, M3) and hindgut (H1, H2, H3).Operational taxonomic units (OTUs) were classified based on 97% sequence similarity. The rarefaction curves for all samples reached the near plateau phase, suggesting good sampling depth.


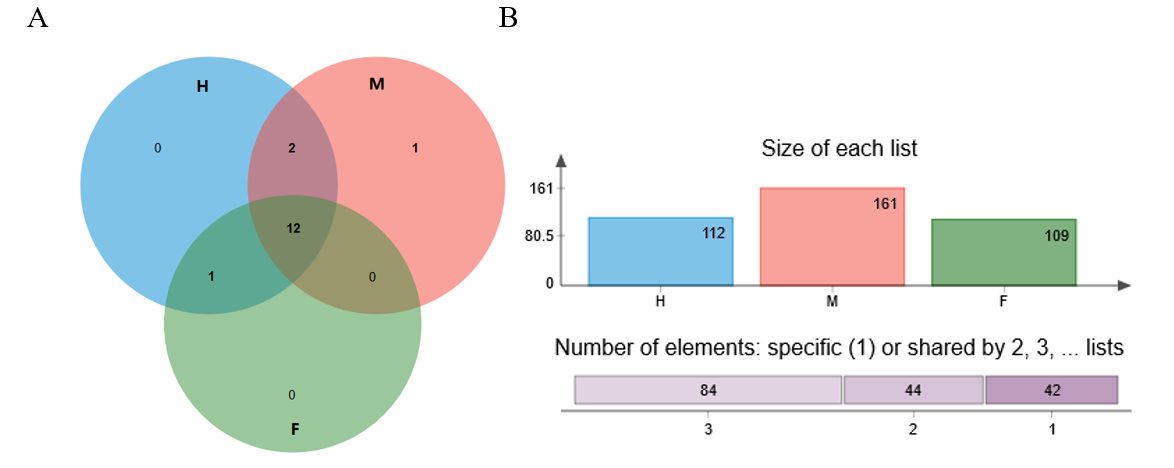


**Fig. S2** Venn diagram of the phylum (A) and the genus level (B) bacterial 16S rRNA gene metagenomic data sets from the digestive tract of crabs at 97% similarity. F, M and H indicate foregut, midgut and hindgut, respectively.


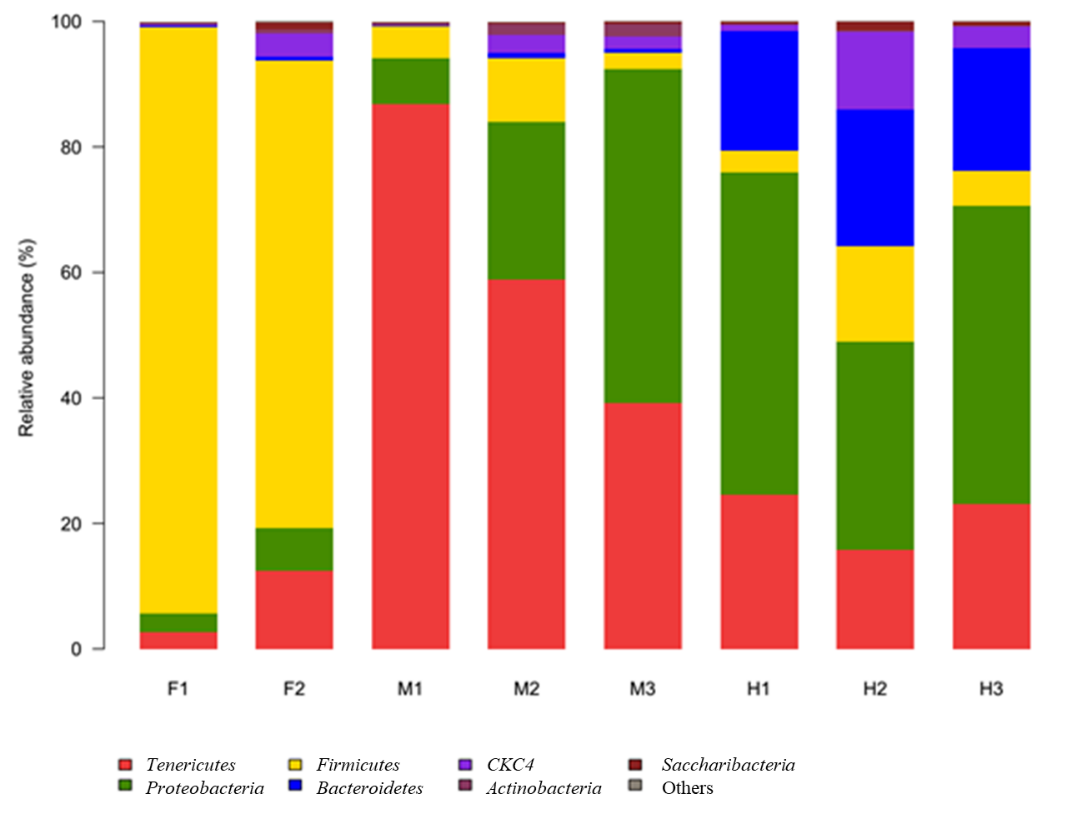


**Fig. S3** The relative abundance of phylum in the composition of digestive tract-associated microbiota of crabs. F: foregut, M: midgut, H: hindgut.


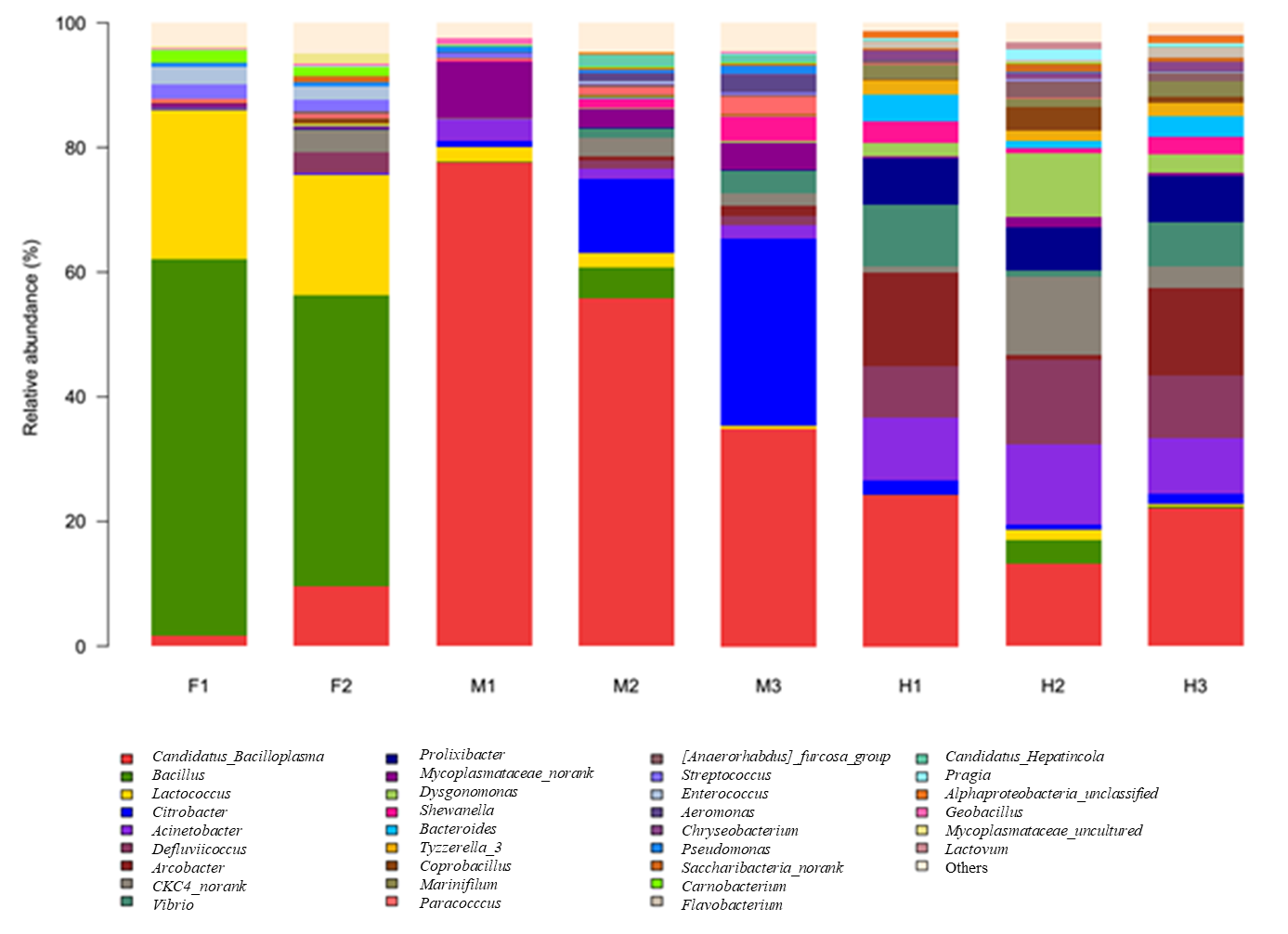


**Fig. S4** The relative abundance of genus level in the composition of digestive tract-associated microbiota of crabs. F: foregut, M: midgut, H: hindgut.


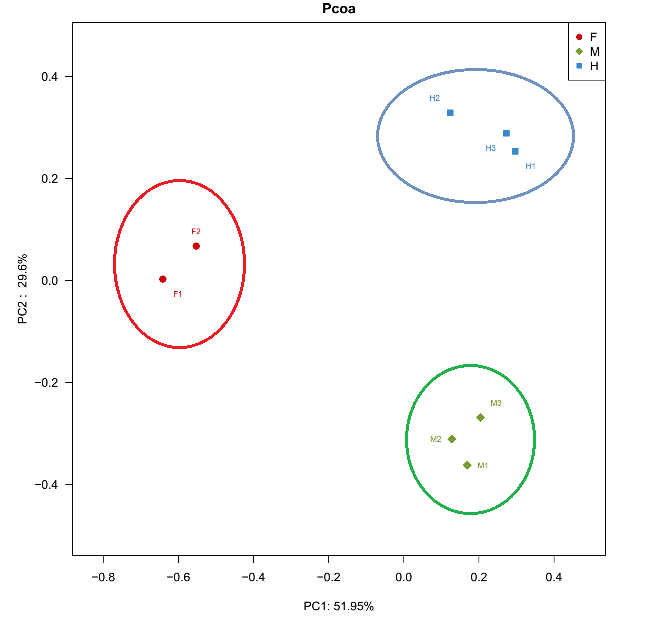


**Fig. S5** Principal coordinate analysis (PCoA) of bacterial communities in the digestive tract samples. F: foregut, M: midgut, H: hindgut.


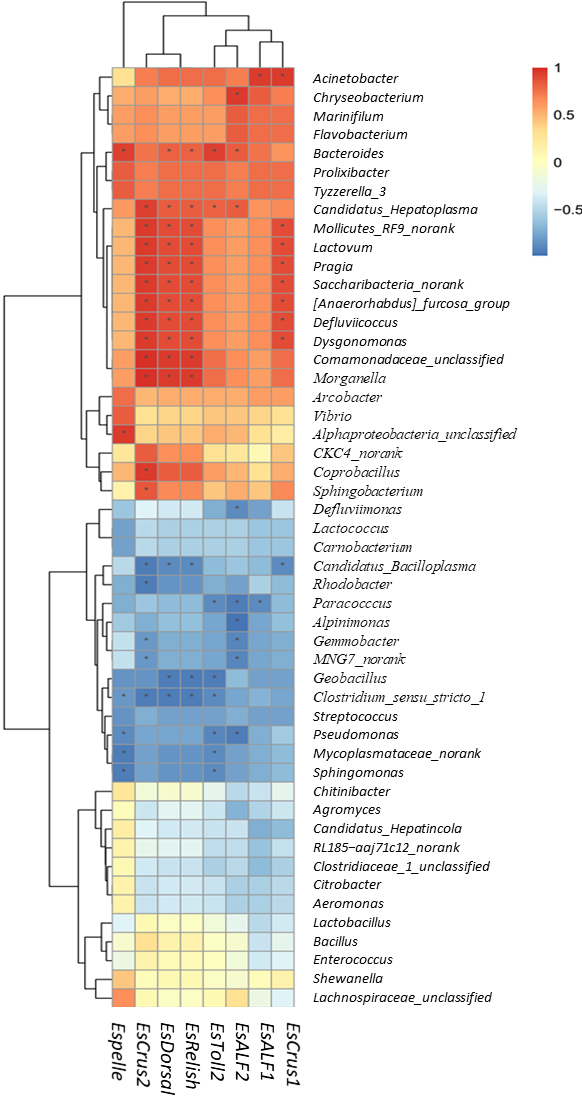


**Fig. S6** Correlation analyses between intestinal microbiota (at the genus level) and the levels of gut immune genes expression. Only the predominant bacterial genera (relative abundance ≥ 1 % in at least one sample) for which abundance was signifcantly associated with eight immune genes expression are presented. R values are correlation coefficient shown in different colors in the figure, the red color represents a positive correlation, the blue color represents a negative correlation. *P* < 0.05 *.

**Table S1** Primers used for quantitative real-time PCR analysis

| **Gene Name** | **Forward and Reverse Sequences** | **Amplicon Size, bp** | **Effeciency %** | **accession numbers** | **Reference** |
| --- | --- | --- | --- | --- | --- |
| *EsToll2* | F: CATACCAGGACGACGAAC | 135 | 101 | KC011816 | This study |
|  | R: AGACATTGAGCGAGGAGA |  |  |  |  |
| *EsPelle* | F: TAAGCCAGCAAACAACGGAGCA | 246 | 95 | KP795393 | Huang et al., 2015 |
|  | R: GAGTCACAGGCAAAGAAGGGGA |  |  |  |  |
| *EsDorsal* | F: CGTCAGCAGCACAGCAGAGAAT | 273 | 105 | KC900086 | Yu et al., 2013 |
|  | R: CCCGTATTTCCTCCCTCAACTTCAG |  |  |  |  |
| *EsRelish* | F: TCTCCCTACTCTGACCATTCC | 163 | 101 | GQ871279 | Li et al., 2013 |
|  | R: TTCCCACCATCTCACTCTTGT |  |  |  |  |
| *EsALF1* | F: GACGCAGGAGGATGCTAAC | 107 | 100 | DQ793214 | Huang et al., 2015 |
|  | R: TGATGGCAGATGAAGGACAC |  |  |  |  |
| *EsALF2* | F: GACCCTTTGCTGAATGCTTGA | 125 | 102 | GU014699 | Huang et al., 2015 |
|  | R: CTGCTCTACAATGTCGCCTGA |  |  |  |  |
| *EsCrus1* | F: GCTCTATGGCGGAGGATGTCA | 115 | 104 | GQ200832 | Huang et al., 2015 |
|  | R: CGGGCTTCAGACCCACTTTAC |  |  |  |  |
| *EsCrus2* | F: GCCCACCTCCCAAACCTAT | 201 | 103 | GQ200833 | Huang et al., 2015 |
|  | R: GCAAGCGTCACAGCAGCACT |  |  |  |  |
| *Es-β-Actin* | F: GCATCCACGAGACCACTTACA | 265 | 102 | HM053699 | Yu et al., 2013 |
|  | R: CTCCTGCTTGCTGATCCACATC |  |  |  |  |

**Primers references**

Huang Y, Chen YH, Zhang YZ, Feng JL, Zhao LL, Zhu HX, Wang W, Ren Q (2015) Identification, characterization, and functional studies of a *Pelle* gene in the Chinese mitten crab, *Eriocheir sinensis*, Fish Shellfish Immunol 45:704-716

Yu AQ, Jin XK, Li S, Guo XN, Wu MH, Li WW, Wang Q (2013) Molecular cloning and expression analysis of a *dorsal* homologue from *Eriocheir sinensis*. Dev Comp Immunol 41:723-727

Li F, Wang L, Zhang H, Zheng P, Zhao J, Qiu L, Zhang Y, Song L (2010) Molecular cloning and expression of a *Relish* gene in Chinese mitten crab *Eriocheir sinensis*. Int J Immunogenet 37:499-508

**Table S2** Effects of foregut (F), midgut (M) and hindgut (H) on the diversity of digestive tract bacterial community at the 3% dissimilarity level. Values shown are means ± SEM, the same column with different letters indicate significantly different (*P*<0.05).

|  | **OTU** | **ACE** | **Chao** | **Shannon** | **Simpson** |
| --- | --- | --- | --- | --- | --- |
| F | 130.33±3.79^ab^ | 147.33±1.53^a^ | 151.00±6.56^a^ | 1.37±0.15^a^ | 0.50±0.09^a^ |
| M | 161.67±29.48^b^ | 177.00±23.81^b^ | 179.33±18.15^b^ | 2.30±0.14^b^ | 0.24±0.04^b^ |
| H | 116.67±8.08^a^ | 140.67±9.45^a^ | 133.33±8.96^a^ | 2.88±0.16^c^ | 0.09±0.02^c^ |
